# The effect of subjective perception of memory and objective cognitive performance on negative affective symptoms in older adults

**DOI:** 10.1186/s12877-026-06974-1

**Published:** 2026-01-15

**Authors:** Eszter Csábi, Dóra Feil, Szilvia Major Sebőkné, Márta Volosin

**Affiliations:** 1 Department of Cognitive and Neuropsychology, Institute of Psychology, University of Szeged, Szeged, Hungary; 2 Institute of Psychology, Metacognition and Behavior Analysis Lab, University of Szeged, Egyetem utca 2, Szeged, H-6722 Hungary

**Keywords:** Subjective memory, Memory concerns, Objective memory abilities, Depression, Anxiety, Stress, Worry of dementia

## Abstract

**Background:**

The relationship between negative affective states and subjective memory is assumed to be bidirectional. Most studies have focused on the effect of negative affective symptoms on subjective memory functioning, whereas the influence of memory functioning on mood is less studied. Therefore, the present study aims to explore the predictive value of perceived memory performance and objective cognitive abilities on negative affective states such as depression, anxiety, and stress.

**Methods:**

A total of 76 older adults participated in the cross-sectional study (mean age = 71.5 years (SD = 4.58), 21 males and 55 females), recruited by convenience and snowball sampling methods. We used the Multifactorial Memory Questionnaire and Cognitive Failures Questionnaire to assess subjective memory functioning, the Montreal Cognitive Assessment to measure objective cognitive performance, and the Depression, Anxiety, Stress-21 Scale to examine negative affective state and stress exposure.

**Results:**

The hierarchical regression models revealed that education (*p* = 0.033, and *p* = 0.044, respectively), cognitive failures (CFQ) (*p* < 0.001), objective cognitive performance (MOCA) (*p* = 0.019, and *p* = 0.024, respectively), and internal strategy use (*p* = 0.015) had a significant predictive value on depression. Education (*p* = 0.037) and cognitive failures (*p* < 0.001) also showed predictive value on anxiety and stress. Furthermore, objective cognitive performance significantly (*p* = 0.045) predicted perceived stress level. In addition, satisfaction with memory was identified as a marginally significant predictor of depression (*p* = 0.078) and stress (*p* = 0.096).

**Conclusion:**

Our results showed that self-perceived memory functioning, memory concerns, and objective memory performance have a predictive value on negative affective symptoms. Thus, objective cognitive abilities and concerns about memory have a significant impact on mental health and well-being. Furthermore, our findings emphasize the importance of assessing negative affective states and perceived health status when evaluating both subjective and objective cognitive performance.

**Supplementary Information:**

The online version contains supplementary material available at 10.1186/s12877-026-06974-1.

## Introduction

Aging is often associated with the increasing level of memory complaints, and with the self-perceived decline of memory functions, while the objectively assessed cognitive abilities appear to be intact. This phenomenon refers to subjective memory complaints (SMCs), and tends to increase in numbers and severity with age [1–4]. Subjective memory complaints refer to the self-experienced worsening of memory functioning compared to previous levels of performance, with normal objective cognition [5]. Normal ageing is related to mild to moderate cognitive deterioration, primarily in prefrontal-guided functions such as memory and executive functions. However, with normal ageing, many of the cognitive functions remain intact until late life. In contrast, pathological ageing, such as Alzheimer’s disease, globally affects brain functions, particularly the medial temporal lobe, resulting in impairment in several domains, disturbing everyday life [6, 7].

SMCs are primarily relevant in older adults and tend to increase in numbers and severity with age [6], but the prevalence of subjective cognitive complaints showed mixed results in the literature. Based on Röhr et al. [8], one-quarter of individuals aged over 60 report self-perceived memory decline. In comparison, Brigola et al. [9] found that approximately 50% of people aged 50 to 59 experience SMC, as do 63% of people aged 80 to 100. Previous studies have also revealed that individuals with higher educational levels are more satisfied with their memory and are more inclined to use external memory aids [10–12]. They are more likely to perceive memory problems, suggesting that subjects with higher education are more sensitive to cognitive decline [13]. These complaints can be present in individuals with cognitive impairment, such as mild cognitive impairment (MCI) or Alzheimer’s disease (AD), as well as in people with no objective cognitive deterioration [14]. Specifically, a recent meta-analysis found that 25% of older adults, who are cognitively healthy but report subjective cognitive complaints, have a twofold risk of progressing to dementia in the next five years [15]. In addition, depression, anxiety, and stress were also found to increase the risk of developing memory concerns [16], therefore, revealing the links between SMCs, cognitive and affective states is essential. Despite that there are several different approaches to the assessment of subjective memory, less is known about the predictive value of self-perceived memory on negative affective states. Consequently, the aim of the present study is to examine how self-perceived and objective cognitive performance are associated with negative affective states.

Although the relationship between the self-perceived memory functioning and objectively measured cognitive abilities is widely studied, studies showed mixed results. Some of them demonstrated a significant relationship between subjective and objective memory performance [17–20]. Indeed, there is also evidence that older adults reporting SMCs tend to experience greater objective cognitive decline and have a high risk of developing pathological cognitive impairments [5, 21–23]. On the other hand, another line of research failed to reveal any association between SMCs and objective cognitive performance [24–26]. The contradictory results may be attributed to the characteristics of the studied population (e.g., age, gender, educational attainment, clinical sample) [27, 28] or methodological variations between the studies, affecting both subjective and objective memory results [5, 29].

Depression is a common mental disorder in older adults although its prevalence differs across various studies, ranging from 8.2 to 63% [30]. According to community-based studies the main risk factors for late life depression are comorbid physical illness, disability, cognitive and functional impairment, increased mortality, decreased social contacts, and previous history of depression [31]. Moreover, stressful life events predict risk for depression and anxiety by disturbing brain mechanisms (e.g., hormones), depleting cognitive capacity, leading to alterations in health behaviour and making individuals vulnerable [32, 33]. Depression, anxiety, and stress can also increase the risk of developing memory concerns [16]. Previous studies revealed that subjective memory evaluation relates to negative affective state rather than cognitive abilities, with no cognitive decline [24, 26, 34]. However, it is still debated whether memory concerns about cognitive decline can cause anxiety and depression, or the negative affective state has an influence on subjective memory functioning [35–37]. For example, a recent study by Giannouli and Tsolaki [38] demonstrated that negative affect had an impact on financial capacity (e.g., knowledge of personal assets, financial decision-making) in older adults, even without depression. On the one hand, complaints of declining memory are often reported among individuals with depressive and anxious symptoms [24, 34, 39, 40]. Studies investigating cognitive functions in patients with depression revealed difficulties in effortful processing, such as sustained attention [41] or the inhibition of emotionally negative distraction [42]. Moreover, verbal learning, executive functions, and memory (e.g., working memory, episodic memory) are commonly impaired domains [43, 44]. There are several explanations for the cognitive deterioration. Firstly, the resource allocation theory supposes that ruminative thinking associated with depression consumes the individual’s limited cognitive resources, reducing the resources available to carry out other cognitive tasks [44, 45]. Secondly, neuroimaging studies found an overlap in neurocircuits involving cognitive processing and emotional regulation. For instance, Frodl et al. [46, 47] demonstrated structural abnormalities in the hippocampus and amygdala in the first diagnosed episode of major depressive disorder. A meta-analysis by MacKinnon et al. [48] showed significant decreases in hippocampal volumes in patients with multiple illness episodes or whose depression was of more than two years duration. In line with these results, Varghese et al. [43] also revealed that individuals with multiple episodes exhibit greater cognitive impairment than individuals with a single episode. Thirdly, according to the social signal transduction theory of depression, the upregulation of proinflammatory cytokines in response to stressful life events can lead to depressive symptoms [49]. Simultaneously, increased inflammation is a contributing factor to neurodegeneration and memory impairments, which are often associated with depression and the ageing process [50].

Some studies also assumed that anxiety and depression precede subjective memory complaints; thus, both depression and anxiety can be risk factors of cognitive decline [51, 52]. Airaksinen et al. [53] revealed that individuals with a higher risk of developing depression showed overactivity in brain regions related to working memory and episodic memory. On the other hand, the perception of memory problems can also lead to symptoms of depression and anxiety [54–56]. Studies focusing on older adults found that lower ratings of subjective memory functioning and higher levels of depression, anxiety, or stress could exacerbate memory-related concerns [16, 54, 57]. The possible explanation of the relationship between affective symptoms and subjective memory complaints appeared to be driven by the fear of dementia [58]. ‘Anticipatory dementia’ is a specific type of health anxiety that is defined by the concern among middle-aged persons or older adults that subjective memory complaints are indicative of future dementia [55, 59]. Thus, negative symptoms may be a reflection of subjective memory complaints with an underlying fear that changes in memory functions and perceptions of memory problems are indicating dementia [54, 58]. Moreover, this is a bidirectional relationship because precipitating worry about dementia can lead to higher levels of depression, anxiety, and stress [16].

Although the link between mood and subjective memory is assumed to be reciprocal [16], most of the previous studies have focused on the effect of negative affective symptoms on subjective memory functioning and memory complaints, and it is less studied how memory functioning influences mood. Therefore, the present study aims to explore the predictive value of perceived memory performance and objective cognitive abilities on negative affective states such as depression, anxiety, and stress. We hypothesise that subjective memory performance and objective cognitive abilities influence negative affective state and stress level.

## Methods

### Participants

In this cross-sectional study we recruited 80 older adults from senior centres using convenience and snowball sampling methods. Based on Steinberg et al. [16], the inclusion criteria were as follows: age 65 or older and no acute or serious medical condition that could affect cognitive functioning. After excluding four individuals from the sample due to mental and behavioural disorders, the final sample consisted of 76 participants. The mean age of the sample was 71.5 years (SD = 4.58), range: 65–80 years, 21 males and 55 females. The average education of the participants was 13.8 years (SD = 3.02; range = 8–20 years). 67.1% (51 participants) of the sample were married, and 32.9% (25 participants) were single. 85.52% of the sample (65 individuals) have one or more medical problems, 17.10% (13 individuals) have none. Appendix 1. represents the medical condition of the sample, based on The International Statistical Classification of Diseases and Related Health Problems 11th Revision (ICD-11) [60]. Following Steinberg et al. [16], after reviewing medical conditions, we revealed a multitude of age-related illnesses, controlled by treatment and not exclusionary.

Before the experiment, all participants provided informed consent. The study was conducted in accordance with the Declaration of Helsinki, and the protocol was approved by the United Ethical Review Committee for Research in Psychology, Hungary (EPKEB; Reference number: 2024-068, 03/07/2024).

### Tasks

#### Multifactorial Memory Questionnaire (MMQ)

We used MMQ to assess different aspects of self-perceived memory functioning and SMCs [61]. The Hungarian version of the questionnaire contains 57 items, but only 55 items are used for calculating the scale score and consist of four subscales [10]. The Satisfaction subscale composed of 18 items indicating the respondent’s satisfaction with their memory functioning. Twenty-two items in the Ability subscale evaluate the most prevalent memory issues throughout the preceding two weeks. Seven items of the External Strategy subscale, such as “make a list” and “use a timer or alarm,” assess how frequently external compensatory techniques are used in daily life. Finally, ten items on the Internal Strategy subscale, such as “create an image” or “create a rhyme”, evaluate the frequency of internal strategies. These latter two subscales simply show how frequently different memory aids are used but do not assess why they are used [10, 61]. Respondents have to indicate each item on a 5-point Likert scale their agreement with the statements or the perceived problems with memory and the frequency of memory aids. Higher scores on MMQ subscales are associated with higher satisfaction with memory functioning, better memory abilities, and a more frequent use of strategies [10, 61]. Cronbach alphas of the subscales ranged from 0.751 to 0.924 (Satisfaction: 0.924, Ability: 0.891, External Strategy: 0.787, Internal Strategy: 0.755), indicating adequate and high internal consistency.

#### Cognitive Failure Questionnaire (CFQ)

CFQ is also used to measure the frequency of everyday cognitive lapses (such as “fail to notice signposts on the road” or “forget appointments”) in the last six months [62, 63]. The questionnaire consists of 25 items, and the respondents have to grade each sentence on a 5-point Likert scale from 0 to 4. Higher points indicate a higher prevalence of cognitive failures [62, 63]. Internal consistency of CFQ was high with Cronbach’s alpha: 0.894.

#### Montreal Cognitive Assessment (MoCA)

We used MoCA for evaluating objective memory performance. It is a 10-minute cognitive screening tool developed to assess cognitive impairment, particularly for detecting MCI. It’s a 30-point scale that assesses various cognitive domains such as memory, working memory, visuospatial skills, language, executive functions, sustained attention, abstract thinking, and orientation. According to the Hungarian version of MoCA, a score of 24 out of 30 is considered to be [64, 65].

#### Depression Anxiety and Stress Scale-21 (DASS-21)

DASS-21 is a short version of a 42-item self-report questionnaire designed to measure the negative emotional states of depression, anxiety, and stress. The questionnaire contains 21 items; each item of the three scales is rated on a 4-point Likert scale from 0 to 3. Participants must indicate how the statements applied to them over the past week. Depression, anxiety, and stress scores are measured by summarizing the scores of the related items. The validation studies classified the resulting ratings as: “normal, mild, moderate, severe, and extremely severe” [66, 67]. The internal consistency of DASS-21 subscales was adequate (Anxiety: Cronbach’s alpha: 0.781; Stress: Cronbach’s alpha: 0.785) and high (Depression: Cronbach’s alpha: 0.836).

### Procedures

Participants were recruited from senior and day care centres affiliated with the university. Those who applied for the study arranged a separate appointment for data collection. Data were collected in a quiet room in the senior centres or the participant’s home. Before testing, each participant received detailed instruction about the aim and procedure of the study and signed the informed consent. Data collection took approximately an hour.

### Statistical analysis

In the first step of the statistical analysis, Spearman’s correlations were used to assess the relationship between subjective memory functioning (CFQ, MMQ) and age, education, gender, the presence of depression, anxiety, and stress (DASS-21), as well as cognitive status (MoCA). The primary variables were education, subjective memory (CFQ, MMQ) and objective cognitive performance (MOCA), as well as the DASS-21 scales. The secondary variables were demographic variables and comorbidities.

In the second step, hierarchical regression models were built using the Enter method, with the outcome variables (dependent variables) being the DASS-21 subscales. The predictors were the significantly correlated variables (education, CFQ, MMQ subscales, and MoCA) with these subjective scales. (The correlations are shown in Appendix 2). To examine the effect of subjective memory functioning and cognitive status on negative affective states, hierarchical linear regression was used. Due to the inconsistent results obtained in subjective memory studies [5, 29] and the differing nature of the two subjective memory questionnaires, we analysed the CFQ and MMQ subscales in separate models. Although both measure subjective memory functioning, the MMQ test was designed specifically for older adults, whereas the CFQ questionnaire is appropriate for individuals of all ages. The MMQ focuses on assessing memory functioning and compensatory strategies, while the CFQ emphasises broader cognitive abilities, such as memory, attention and executive functions [61, 62].

Hierarchical regression models were applied to measure constructs both by individual predictors and by the combined contribution of sequentially added sets of predictors [68]. The baseline models included depression, anxiety, and stress as dependent variables separately. At the first level, average education, the second level, the scores of subjective memory tests (CFQ, MMQ), and at the third level, the score of the cognitive status (MoCA) were submitted as predictors. For each model, the level of autocorrelation was calculated by Durbin-Watson (DW) tests, and the multicollinearity for the predictors was assessed by the variation inflation index (VIF). We considered VIF as satisfactory below 3 [69], and recognized DW values as satisfactory between 1.5 and 2.5. We conducted statistical analysis in Jamovi (version 2.2.5) [70]. Because no a-priori sample size calculation was applied, in order to reveal the sensitivity of our analyses on the present sample size, post-hoc sensitivity analysis was implemented using G*Power 3.1.9.4 [71].

## Results

### Descriptive statistics and correlations

Table 1. shows the means, medians, and standard deviations for each variable, along with the minimum to maximum range across the whole sample. The correlation between negative affective state, subjective memory functioning, cognitive status, and demographic variables demonstrated in Appendix 2. In order to shed more light on the nature of subjective memory functioning, we also examined the association between CFQ, MMQ subscales, demographic variables, and cognitive status. See in Appendix 3.

**Table 1:** Descriptive statistics of the sample (*N* = 76)

	Mean	Median	Standard deviation	Minimum	Maximum
CFQ	26.6	25	12.1	0	58
MMQ_Satisfaction	50.3	50.5	14.4	20	72
MMQ_Ability	60.6	61	10.3	37	80
MMQ_Int.Str.	7.37	7	5.56	0	24
MMQ_Ext. Str.	12.5	12	6.76	0	28
DASS-21_Depression	3.58	3	3.66	0	17
DASS-21_Anxiety	3.71	3	3.56	0	16
DASS-21_Stress	5.18	5	3.81	0	16
MOCA	23.9	24	3.42	15	30

Note. *MMQ – Int. Str*.= Multifactorial Memory Questionnaire Internal Strategy subscale, *MMQ -Ext-. = Str.* Multifactorial Memory Questionnaire External Strategy subscale, *MoCA=* Montreal Cognitive Assessment total score

### Hierarchical linear regression models

#### The effect of subjective memory functioning on depression

##### DASS-21 Depression subscale and Cognitive Failure Questionnaire

The model had three levels: the first contained education, the second was the CFQ score, and the third was the MoCA total score as predictors. The dependent variable was the DASS-21 Depression subscale. The Durbin-Watson test revealed the lack of autocorrelation (Model 1: 1.84, Model 2: 2.12, and Model 3: 2.15). In Model 1, the predictor explained 17.5% of the variance (*R*^*2*^_*adj*_ = 16.4%; *F*(1, 74) = 15.7, *p* < 0.001), Model 2 explained 32.8% of the variance (*R*^*2*^_*adj*_ = 30.1%; F(2, 73) = 17.8, *p* < 0.001) and Model 3 explained 37.8% of the variance (*R*^*2*^_*adj*_ = 35.2%; F(3, 72) = 14.6, *p* < 0.001). The differences between Model 1 and Model 2 (*ΔR*^*2*^ = 15.3%, *F*(1,73) = 16.68, *p* < 0.001, Cohen’s f^2^ = 0.227) were significant as well as between Model 2 and Model 3 (*ΔR*^*2*^ = 4.9%, *F*(1,72) = 5.75, *p* = 0.016, Cohen’s f^2^ = 0.080). In the final model, Education (β = -0.240), CFQ score (β = 0.420), and MoCA total score (β = -0.263) had a significant predictive value on depression. Entering the MoCA total score in the model weakened the effect of education, which still remained significant but had no impact on the effect of CFQ. The results are seen in Table 2.

**Table 2:** Results of the hierarchical linear regression model where the outcome variable is the DASS-21 depression subscale

		B	SE	t	*p*	ß	VIF
Model 1	Intercept	10.53	1.79	5.86	< 0.001		
	Education	− 0.506	0.128	-3.96	< 0.001	-0.403	1.00
Model 2	Intercept	6.735	1.87	3.58	0.001		
	Education	-0.459	0.11	-3.94	< 0.001	-0.379	1.01
	CFQ	0.119	0.02	4.08	< 0.001	0.394	1.01
Model 3	Intercept	10.910	2.52	4.33	< 0.001		
	Education	-0.290	0.13	-2.18	0.033	-0.240	1.40
	CFQ	0.127	0.02	4.47	< 0.001	0.420	1.02
	MoCA	-0.2821	0.11	-2.40	0.019	-0.263	1.39

Note. *CFQ* = Cognitive Failure Questionnaire, *MoCA* = Montreal Cognitive Assessment total score

##### DASS-21 Depression subscale and Multifactorial Memory Questionnaire

The model had three levels: the first contained education, the second was MMQ subscales scores, and the third was the MoCA total score as predictors. The outcome variable was the DASS-21 Depression subscale. The Durbin-Watson test revealed the lack of autocorrelation (Model 1: 1.84, Model 2: 2.11, and Model 3: 2.15). In Model 1, the predictor explained 17.5% of the variance (*R*^*2*^_*adj*_ = 16.4%; *F*(1, 74) = 15.7, *p* < 0.001), Model 2 explained 38% of the variance (*R*^*2*^_*adj*_ = 34.5%; F(4, 71) = 10.9, *p* < 0.001) and Model 3 explained 42.3% of the variance (*R*^*2*^_*adj*_ = 38.2%; F(5, 70) = 10.3, *p* < 0.001). The differences between Model 1 and Model 2 (*ΔR*^*2*^ = 20.51%, *F*(3,71) = 7.82, *p* < 0.001, Cohen’s f^2^ = 0.330) were significant as were those between Model 2 and Model 3 (*ΔR*^*2*^ = 4.37%, *F*(1,70) = 5.30, *p* = 0.024, Cohen’s f^2^ = 0.074). MMQ Internal Strategy (β = 0.275) and MoCA total score (β = -0.249) had significant predictive value on depression, while education (β = -0.196) and MMQ Satisfaction (β = -0.235) showed a trend for predictive value. Entering the MoCA total score in the model weakened the effect of education but improved the effect of MMQ Satisfaction subscales. The results are presented in Table 3.

**Table 3:** Results of the hierarchical linear regression model where the outcome variable is the DASS-21 depression subscale

		B	SE	t	*p*	ß	VIF
Model 1	Intercept	10.532	1.79	5.86	< 0.001		
	Education	− 0.506	0.128	-3.96	< 0.001	-0.418	1.00
Model 2	Intercept	11.909	3.46	3.43	< 0.001		
	Education	-0.400	0.11	-3.59	0.001	-0.331	1.11
	MMQ - S	-0.045	0.03	-1.34	0.185	-0.178	2.03
	MMQ - A	-0.031	0.05	-0.62	0.535	-0.089	2.35
	MMQ – Int. Str.	0.189	0.07	2.53	0.013	0.287	1.47
Model 3	Intercept	16.299	3.86	4.212	< 0.001		
	Education	-0.237	0.13	-1.75	0.084	-0.196	1.53
	MMQ - S	-0.059	0.03	-1.786	0.078	-0.235	2.11
	MMQ - A	-0.023	0.04	-0.466	0.643	-0.065	2.36
	MMQ – Int. Str.	0.181	0.07	2.504	0.015	0.275	1.47
	MoCA	-0.266	0.11	-2.30	0.024	-0.249	1.43

Note. *MMQ - S* = Multifactorial Memory Questionnaire Satisfaction subscale, *MMQ - A* = Multifactorial Memory Questionnaire Ability subscale, *MMQ – Int. Str.=* Multifactorial Memory Questionnaire Internal Strategy subscale, *MoCA =* Montreal Cognitive Assessment total score

#### The effect of subjective memory functioning on anxiety

##### DASS-21 Anxiety Subscale and Cognitive Failure Questionnaire

The hierarchical linear regression model had three levels: the first contained education, the second was CFQ score, and the third was MoCA total score as predictors. The dependent variable was the DASS-21 Anxiety subscale. The Durbin-Watson test revealed the lack of autocorrelation (Model 1: 1.92, Model 2: 2.23, and Model 3: 2.20). In Model 1, the predictor explained 14.1% of the variance (*R*^*2*^_*adj*_ = 12.9%; *F*(1, 74) = 12.2, *p* < 0.001), Model 2 explained 33.3% of the variance (*R*^*2*^_*adj*_ = 31.4%; F(2, 73) = 18.2, *p* < 0.001) and Model 3 explained 35.7% of the variance (*R*^*2*^_*adj*_ = 33.1%; F(3, 72) = 13.3, *p* < 0.001). Model 1 and Model 2 showed significant differences (*ΔR*^*2*^ = 19.015%, *F*(1,73) = 20.95, *p* < 0.001, Cohen’s f^2^ = 0.287), and a trend was found between Model 2 and Model 3 (*ΔR*^*2*^ = 2.48%, *F*(1,72) = 2.77, *p* = 0.100, Cohen’s f^2^ = 0.037). Education (β = -0.234) and CFQ score (β = 0.458) had a significant predictive value on anxiety., MoCA total score (β = -0.214) had no predictive value on anxiety. The results are seen in Table 4.

**Table 4:** Results of the hierarchical linear regression model where the outcome variable is the DASS-21 anxiety subscale

					B			SE		t	*p*		
Model 1		Intercept	9.792		1.78		5.48	< 0.001					
		Education	− 0.442		0.127		-3.49	0.002		-0.376			1.00
Model 2		Intercept	5.664		1.82		3.11	0.003					
		Education	-0.391		0.11		-3.46	0.001		-0.332			1.01
		CFQ	0.129		0.02		4.58	< 0.001		0.440			1.01
Model 3		Intercept	8.534		2.49		3.42	0.001					
		Education	-0.275		0.13		-2.09	0.040		-0.234			1.40
		CFQ	0.135		0.02		4.80	< 0.001		0.458			1.02
		MoCA	-0.193		0.11		-1.67	0.100		-0.186			1.39

Note. *CFQ* = Cognitive Failure Questionnaire, *MoCA* = Montreal Cognitive Assessment total score

##### DASS-21 Anxiety subscale and Multifactorial Memory Questionnaire

The model had three levels: the first contained education, the second was MMQ subscales scores, and the third was the MoCA total score as predictors. The outcome variable was the DASS-21 Depression subscale. The Durbin-Watson test revealed the lack of autocorrelation (Model 1: 1.92, Model 2: 2.06, and Model 3: 2.05). In Model 1, the predictor explained 14.1% of the variance (*R*^*2*^_*adj*_ = 12.9%; *F*(1, 74) = 12.15, *p* = 0.002), Model 2 explained 26.2% of the variance (*R*^*2*^_*adj*_ = 22%; F(4, 71) = 6.30, *p* < 0.001) and Model 3 explained 27.4% of the variance (*R*^*2*^_*adj*_ = 22.3%; F(5, 70) = 5.29, *p* < 0.001). The differences between Model 1 and Model 2 (*ΔR*^*2*^ = 12.09%, *F*(3,75) = 3.71, *p* = 0.013, Cohen’s f^2^ = 0.116) were significant. There were no significant differences between Model 2 and Model 3 (*ΔR*^*2*^ = 1.25%, *F*(1,70) = 1.20, *p* = 0.27, Cohen’s f^2^ = 0.003). Education (β = -0.267) showed predictive value on anxiety. Entering the MoCA total score into the model weakened the effect of education and MMQ Internal Strategy. The results are seen in Table 5.

**Table 5:** Results of the hierarchical linear regression model where the outcome variable is the DASS-21 anxiety subscale

		B	SE	t	*p*	ß	VIF
Model 1	Intercept	9.792	1.78	5.48	< 0.001		
	Education	− 0.442	0.127	-3.49	< 0.001	-0.376	1.00
Model 2	Intercept	13.070	3.68	3.550	0.001		
	Education	-0.399	0.12	-3.159	0.002	-0.339	1.11
	MMQ - S	0.006	0.03	0.175	0.086	0.254	2.03
	MMQ - A	-0.082	0.05	-1.528	0.131	-0.238	2.35
	MMQ – Int. Str.	0.115	0.07	1.462	0.148	0.180	1.47
Model 3	Intercept	15.352	4.22	3.63	< 0.001		
	Education	-0.315	0.14	-2129	0.037	-0.267	1.53
	MMQ - S	-0.001	0.03	-0.033	0.974	-0.004	2.11
	MMQ - A	-0.078	0.05	-1.442	0.154	-0.225	2.36
	MMQ – Int. Str.	0.111	0.07	1.411	0.163	0.174	1.47
	MoCA	-0.138	0.12	-1.096	0.227	-0.133	1.43

Note. *MMQ - S* = Multifactorial Memory Questionnaire Satisfaction subscale, *MMQ - A*  = Multifactorial Memory Questionnaire Ability subscale, *MMQ – Int. Str.* = Multifactorial Memory Questionnaire Internal Strategy subscale, *MoCA* = Montreal Cognitive Assessment total score

#### The effect of subjective memory functioning on stress level

##### DASS-21 Stress subscale and Cognitive Failure Questionnaire

The hierarchical linear regression model had three levels: the first contained education, the second was CFQ score, and the third was MoCA total score as predictors. The dependent variable was the DASS-21 Stress subscale. The Durbin-Watson test revealed the lack of autocorrelation (Model 1: 1.73, Model 2: 1.92, and Model 3: 1.92). In Model 1, the predictor explained 13.6% of the variance (*R*^*2*^_*adj*_ = 12.4%; *F*(1, 74) = 11.6, *p* = 0.001), Model 2 explained 31.7.8% of the variance (*R*^*2*^_*adj*_ = 29.8%; F(2, 73) = 16.9, *p* < 0.001) and Model 3 explained 36% of the variance (*R*^*2*^_*adj*_ = 33.3%; F(3, 72) = 13.5, *p* < 0.001). The differences between Model 1 and Model 2 (*ΔR*^*2*^ = 18.14%, *F*(1,7) = 19.39, *p* < 0.001, Cohen’s f^2^ = 0.265) were significant, as were the differences between Model 2 and Model 3 (*ΔR*^*2*^ = 4.25%, *F*(1,72) = 4.78, *p* = 0.032, Cohen’s f^2^ = 0.023). CFQ score (β = 0.452), education (β = -0.197) and MoCA total score (β = -0.243) had a significant predictive value on stress. Entering the MoCA total score in the model weakened the effect of education but had no impact on the effect of CFQ. The results are seen in Table 6.

**Table 6:** Results of the hierarchical linear regression model where the outcome variable is the DASS-21 stress subscale

		B	SE	t	*P*	ß	VIF
Model 1	Intercept	11.566	1.91	6.03	< 0.001		
	Education	− 0.64	0.136	-3.41	< 0.001	-0.368	1.00
Model 2	Intercept	7.267	1.97	3.68	< 0.001		
	Education	-0.411	0.12	-3.36	0.001	-0.326	1.01
	CFQ	0.113	0.03	4.40	< 0.001	0.408	1.01
Model 3	Intercept	11.292	2.66	4.24	< 0.001		
	Education	-0.249	0.14	-1.77	0.082	-0.197	1.40
	CFQ	0.142	0.03	4.74	< 0.001	0.452	1.02
	MoCA	-0.271	0.12	-2.19	0.032	-0.243	1.39

Note. *CFQ = *Cognitive Failure Questionnaire, *MoCA = *Montreal Cognitive Assessment total score

##### DASS-21 Stress subscale and Multifactorial Memory Questionnaire

The hierarchical linear regression model had three levels: the first contained education, the second was MMQ subscales scores, and the third was the MoCA total score as predictors. The outcome variable was the DASS-21 Stress subscale. The Durbin-Watson test revealed the lack of autocorrelation (Model 1: 1.73, Model 2: 1.90, and Model 3: 1.91). In Model 1, the predictor explained 13.6% of the variance (*R*^*2*^_*adj*_ = 124%; *F*(1, 74) = 9.17, *p* = 0.001), Model 2 explained 34,8% of the variance (*R*^*2*^_*adj*_ = 30.2%; F(5, 70) = 7.248, *p* < 0.001) and Model 3 explained 38.6% of the variance (*R*^*2*^_*adj*_ = 33.2%; F(6, 69) = 7.21, *p* < 0.001). The differences between Model 1 and Model 2 (*ΔR*^*2*^ = 21.26%, *F*(4,70) = 5.71, *p* < 0.001, Cohen’s f^2^ = 0.325) were significant. Model 2 and Model 3 also demonstrated significant differences (*ΔR*^*2*^ = 3.72%, *F*(1,69) = 4.18, *p* = 0.045, Cohen’s f^2^ = 0.061). MoCA total score (β = -0.230) showed a significant predictive value on stress. MMQ Satisfaction demonstrated a trend for predictive value (β = -0.232). Entering MMQ subscales in the model weakened the effect of education. Furthermore, entering the MoCA total score into the model eliminated the effect of education and weakened the effect of MMQ Internal and External Strategy, but improved the effect of MMQ Satisfaction subscale. The results are presented in Table 7.

**Table 7:** Results of the hierarchical linear regression model where the outcome variable is the DASS-21 stress subscale

		B	SE	t	*p*	ß	VIF
Model 1	Intercept	11.566	1.91	6.03	< 0.001		
	Education	− 0.464	0.135	-3.41	0.001	-0.368	1.00
Model 2	Intercept	14.813	3.74	3.95	< 0.001		
	Education	-0.378	0.12	-2.91	0.005	-0.300	1.14
	MMQ - S	-0.047	0.03	-1.30	0.196	-0.180	2.05
	MMQ - A	-0.055	0.05	-1.01	0.315	-0.149	2.35
	MMQ – Int. Str.	0.021	0.06	0.35	0.727	0.037	1.26
	MMQ – Ext. Str.	0.146	0.08	0.70	0.092	0.213	1.69
Model 3	Intercept	19.023	4.20	4.52	< 0.001		
	Education	-0.222	0.14	-1.50	0.176	-0.133	1.55
	MMQ - S	-0.061	0.03	-1.68	0.096	-0.232	2.12
	MMQ - A	-0.047	0.05	-0.87	0.383	-0.127	2.36
	MMQ – Int. Str.	0.138	0.08	1.64	0.105	0.201	1.26
	MMQ – Ext. Str.	0.023	0.05	0.39	0.696	0.041	1.69
	MoCA	-0.2456	0.12	-2.04	0.045	-0.230	1.43

Note. *MMQ - S* = Multifactorial Memory Questionnaire Satisfaction subscale, *MMQ - A* = Multifactorial Memory Questionnaire Ability subscale, *MMQ – Int. Str.* = Multifactorial Memory Questionnaire Internal Strategy subscale, *MMQ – Ext. Str.* = Multifactorial Memory Questionnaire External Strategy subscale, *MoCA* = Montreal Cognitive Assessment total score

Post-hoc sensitivity analysis indicated that final models with the present sample size were able to detect effects of Cohen’s *f*^*2*^ = 0.151–0.196 (*η*^*2*^ = 0.131–0.163) with 80% power. This suggests that our analyses were sensitive enough to detect at least medium to large effects, that is, small to medium effects might have remained undetected [72].

## Discussion

The aim of the current study was to examine the predictive value of subjective and objective memory performance on negative affective states in older adults. We found that CFQ correlated positively with depression, anxiety, and stress, and both CFQ and MoCA had a significant predictive value on the levels of depression, and stress. Our analysis also revealed that MMQ Satisfaction and MMQ Ability subscales demonstrated significant negative correlation with depression, anxiety, and stress. Positive association was found between MMQ Internal Strategy and depression, anxiety, and stress, and the MMQ External Strategy subscale also showed positive correlation with stress. The hierarchical linear regression models revealed that MMQ Internal Strategy and MoCA had a significant predictive value on depression and stress, but not on anxiety. While entering MoCA into the models improved the predictive value of MMQ Satisfaction on the levels of depression and stress, it weakened the predictive value of MMQ Internal and External Strategy in the case of stress and anxiety. Furthermore, the MoCA total score and education showed significant negative correlation with depression, anxiety, and stress. However, entering the MoCA total score into the hierarchical regression model weakened the effect of education on a negative affective state and eliminated the predictive value of education on stress.

We revealed that self-perceived memory performance and objective memory performance have a predictive value on negative affective states. Individuals who are more satisfied with their memory have better objective memory performance, fewer SMCs, and a lower level of depression, anxiety, and stress. These findings are in line with previous studies that found that perception of memory functioning has a crucial role in psychological well-being [54]. Higher levels of SMCs are associated with more depressive symptoms and higher stress exposure [54, 55, 73]. Studies investigating the association between memory concerns and negative affective state assumed that this relationship are based on the worry about cognitive decline [54, 55, 58, 59], which is one of the greatest concerns associated with aging. Particularly, when perceiving one’s memory functioning as poorer compared to their peers. Based on Mogle et al. [73] results, age-anchored and self-anchored comparison activates social threat (e.g. dementia, loss of independence, personal and environmental reality, or individuals’ identity) mechanism but may not be related to actual performance deterioration. They also found that better ratings of subjective memory were associated with higher levels of life satisfaction and fewer depressive symptoms [73]. Consistent with these results, Norman et al. [58] also revealed that fear of Alzheimer’s disease was the strongest predictor of SMCs, even after accounting for age and education. In their study, nearly half of their sample reported being very concerned or somewhat concerned about developing AD. The authors also assumed that one aspect of fear of AD may be related to thoughts of uncontrollability associated with perceived disease-related memory changes [58]. Another possible explanation for the worry of dementia could be the negative socio-cultural framework associated with AD patients and their family members regarding the cognitive, emotional, and behavioural implications of the disease (for a review, see Giannouli [74]). The bidirectional nature of the relationship between negative symptoms and SMCs indicates that a higher level of anxiety, including fear of dementia, may enhance the awareness of age-related memory changes that may result in SMCs [58, 75]. A study by Verhaeghen et al. [76] also demonstrated that higher perceptions of memory problems may lead to increased memory-related anxiety, which may further lead to an increase in perceived seriousness of memory problems. Indeed, Begum et al. [77] found that when individuals were asked to rank a list of health-related symptoms in terms of their perceived level of importance, participants who ranked subjective cognitive symptoms as highly important also had higher levels of depressive symptoms. Tandem with these findings, longitudinal studies demonstrated that subjective cognitive complaints at baseline were associated with depressive symptoms in subjects without cognitive decline over time, including a greater risk of developing depression after a 2 and 10-years follow-up period [78–80].

Negative cognitive biases are also common in depression [81, 82] and patients with depression tend to underestimate their performance. There is evidence that negative symptoms negatively affect self-perceived cognition in bipolar disorder and major depressive disorder [83]. Negatively formed self-perception could be an explanation for why patients with major depressive disorder commonly complain about their cognitive functions in everyday life. In contrast, their performance on objective cognitive tasks is not severely impaired [42]. Another possible explanation of the discrepancy between objective performance and subjective evaluation of cognitive functions in depressed patients is the absence of motivation. They might be motivated to participate in cognitive testing in a laboratory setting, but they have difficulty finding motivation in everyday life. Furthermore, patients’ complaints may influence family members’ impressions and, therefore, do not necessarily reflect the patient’s objective everyday performance [42].

Our data showed that the frequency of internal strategy use was associated with depression, anxiety, and stress. Moreover, internal strategy use had a predictive value on depression and stress. These results are in tune with previous literature showing that individuals with more negative symptoms and higher levels of stress exposure tend to utilize more internal and external memory aids. These findings may be explained by the fact that negative affect was associated with increased self-monitoring and error awareness [84], and these traits might also lead to greater reported SMCs and more frequent use of internal strategies [10]. Additionally, it is feasible that higher level of anxiety can cause a false impression about memory capacities and lead to higher vulnerability and poorer coping mechanisms under stressful circumstances [58, 85]. Therefore, they might feel the need to use more compensatory strategies. Consistent with these findings, older adults tend to use more memory strategies to improve everyday memory [86], particularly in higher level of SMCs and poorer objective memory performance [85]. In line with these results, fewer memory concerns and better cognitive functioning leads to using compensatory strategies less frequently [85, 87]. This observation may explain our results that entering MoCA into the regression model weakened the predictive value of the frequency of external and internal strategy use. Lin et al. [85] also revealed that a lower level of depression and increased quality of life were associated with less investment in strategies due to satisfaction with memory functioning.

Finally, a negative correlation was found between education and negative affective state, and education has a significant predictive value on negative symptoms as well. However, entering the MoCA total score into the hierarchical regression model weakened the effect of education on depression and anxiety, and eliminated the predictive value of education on stress. This result is consistent with previous studies indicating that individuals with a higher level of education are more perceptive of the quality of their cognitive performance as well as of the potential signs of cognitive decline [10, 13, 63].

Our study has some limitations that should be noted. The main limitation of the study is its relatively small sample size, which leads to the lack of power, and makes it difficult to detect subtle relations and effects. The second limitation is the cross-sectional approach of the study, which prevents us from examining the temporal ordering of these relationships and their long-term effects. Future studies should focus on the long-term relationship between negative symptoms and subjective memory functioning. Furthermore, we cannot rule out the possible impact of the individual’s biased self-perception and the potential influence of comorbid disease and medication. In addition, a major drawback of the linear regression models is that although they are suitable to measure the additive effect of predictors, these are direct effects only; therefore, more complex relationships between variables might remain undetected [68].

## Conclusion

In conclusion, we found that subjective cognition and objective memory performance have a predictive value on negative affective states such as depression, anxiety, and stress. Our results highlight that the deterioration of cognitive functions and the underestimation of one’s own abilities could be a risk factor for late-life depression, increased symptoms of anxiety and a higher level of perceived stress. These findings underpin the importance of considering both subjective and objective cognition when addressing depression in older adults. It could be important for treatment approaches for older adults to prevent or treat the development of negative perceptions of their own ability and related distress, which can exacerbate SMCs, decrease objective memory performance, and negatively impact affective state.

## Supplementary Information

Supplementary Material 1

Supplementary Material 2

## Data Availability

The datasets generated and analysed during the current study are available from the corresponding author on reasonable request.

## Appendix 1

**Table:** Medical condition of the sample (N = 76)

Medical condition	Frequency/number	Percentage
Disease of circulatory system	46	60.5%
Endocrine, nutrition, and metabolic disease	11	14.5%
Diseases of the musculoskeletal system and connective tissue	4	5.3%
Neuroplasm	1	1.3%
Diseases of the skin and subcutaneous tissue	1	1.3%
Diseases of the genitourinary system	1	1.3%
Diseases of the eye and adnexa	1	1.3%

## Appendix 2

Correlation between negative affective state, subjective memory functioning, cognitive status, and demographic variables

DASS-21 Depression subscale showed a significant moderate negative correlation with education, MMQ Satisfaction subscale, MMQ Ability subscale, and MoCA total score. The CFQ and MMQ Internal Strategy subscale also demonstrated a significant, moderate positive association with depression. We failed to find a correlation between depression and age and the MMQ External Strategy subscale.

We revealed a moderate negative correlation between the DASS-21 Anxiety subscale and education, MMQ Satisfaction, and MMQ Ability subscales. The MoCA total score also demonstrated a significantly weak negative association with anxiety. A significant moderate positive correlation was present between anxiety and CFQ, and the MMQ Internal Strategy subscale was also correlated positively with the Anxiety subscale. There was no correlation between anxiety, age, and the MMQ External Strategy subscale.

We found a significant moderate negative association between the DASS-21 Stress subscale and MMQ Satisfaction, MMQ Ability subscales, and MoCA total score also showed a significant negative correlation with stress. Education also demonstrated a weak negative correlation with stress. CFQ, MMQ Internal Strategy were correlated positively with the Stress subscale. No correlation was found between stress and age and MMQ External Strategy subscale. Results are presented in Table 2.

**Table:** Correlation between mood, demographic variables, subjective memory complaints, cognitive status

	DASS-21 Depression	DASS-21 Anxiety	DASS-21 Stress
Age	-0.056	-0.101	-0.083
Education	-0.446***	-0.370**	-0.373***
CFQ	0.460***	0.446***	0.437***
MMQ Satisfaction	-0.497***	-0.375***	-0.468***
MMQ Ability	-0.397***	-0.365**	-0.392***
MMQ External Strategy	0.016	0.019	0.130
MMQ Internal Strategy	0.448***	0.313***	0.400***
MoCA total score	-0.422***	-0.238*	-0.305*

*df* 78, *CFQ* Cognitive Failure Questionnaire, *MoCA* Montreal Cognitive Assessment, *DASS-21* Depression, Anxiety, Stress Scale-21, *MMQ * Multifactorial Memory Questionnaire, *External Str.* External Strategy subscale, *Internal Str.* Internal Strategy subscale

Note. *p < 0.05, p** < 0.01, p*** < 0.001

## Appendix 3

Correlation between subjective, objective memory and demographic variables

Significant weak negative correlation was present between CFQ and age (r(74) = -0.286, p = 0.012), although age did not correlate with any of the MMQ subscales (MMQ Satisfaction: r(74) = 0.173, p = 0.136, MMQ Ability: r(74) = 0.135, p = 0.246, MMQ Internal Strategy: r(74) = 0.033, p = 0.777, MMQ External Strategy: r(74) = 0.024, p = 0.837). Gender did not show any significant correlation with CFQ (r(74) = -0.028, p = 0.809) and MMQ subscales (MMQ Satisfaction: r(74) = 0.051, p = 0.662, MMQ Ability: r(74) = 0.171, p = 0.139, MMQ Internal Strategy: r(74) = -0.040, p = 0.729, MMQ External Strategy: r(74) = 0.085, p = 0.467). A weak negative correlation were found between education and MMQ Internal Strategy (r(74) = -0.229, p = 0.047). We failed to find association between education and CFQ (r(74) = -0.073, p = 0.528) and MMQ subscales (MMQ Satisfaction: r(74) = 0.141, p = 0.225, MMQ Ability: r(74) = -0.007, p = 0.955, MMQ External Strategy: r(74) = 0.037, p = 0.748).

MoCA did not correlated with CFQ (r(74) = 0.048, p = 0.678) and none of the MMQ subscales (MMQ Satisfaction: r(74) = -0.039, p = 0.741, MMQ Ability: r(74) = -0.041, p = 0.725, MMQ Internal Strategy: r(74) = -0.135, p = 0.3245, MMQ External Strategy: r(74) = 0.084, p = 0.473).

## Abbreviations

SMC: Subjective Memory Complaints
MCI: Mild Cognitive Impairment
AD: Alzheimer’s Disease
ICSD-11: International Statistical Classification of Diseases and Related Health Problems 11th Revision
MMQ: Multifactorial Memory Questionnaire
CFQ: Cognitive Failure Questionnaire
MoCA: Montreal Cognitive Assessment
DASS-21: Depression. Anxiety and Stress Scale-21
DW: Durbin Watson test
VIF: Variation Inflation Index
