# Supplementary material for: The effect of subjective perception of memory and objective cognitive performance on negative affective symptoms in older adults
Source: BMC Geriatr. 2026 Jan 15;26:195. doi: 10.1186/s12877-026-06974-1 (PMC12895808; doi:10.1186/s12877-026-06974-1)
Supplement: Supplementary file 1 — Supplementary Material 1 [file 12877_2026_6974_MOESM1_ESM.doc]

Appendix 1. Medical condition of the sample (N = 80)

| **Medical condition** | **Frequency/number** | **Percentage** |
| --- | --- | --- |
| Disease of circulatory system | 49 | 61.3% |
| Endocrine, nutrition, and metabolic disease | 12 | 15% |
| Disease of the nervous system | 4 | 5% |
| Diseases of the musculoskeletal system and connective tissue | 4 | 5% |
| Neuroplasm | 1 | 1.3% |
| Mental and behavioural disorder | 1 | 1.3% |
| Diseases of the skin and subcutaneous tissue | 1 | 1.3% |
| Diseases of the genitourinary system | 1 | 1.3% |
| Diseases of the eye and adnexa | 1 | 1.3% |

Appendix 2. Correlation between negative affective state, subjective memory functioning, cognitive status, and demographic variables

DASS-21 Depression subscale showed a significant moderate negative correlation with education, MMQ Satisfaction subscale, MMQ Ability subscale, and MoCA total score. The CFQ and MMQ Internal Strategy subscale also demonstrated a significant, moderate positive association with depression. We failed to find a correlation between depression and age and the MMQ External Strategy subscale.

We revealed a moderate negative correlation between the DASS-21 Anxiety subscale and education, MMQ Satisfaction, and MMQ Ability subscales. The MoCA total score also demonstrated a significantly weak negative association with anxiety. A significant moderate positive correlation was present between anxiety and CFQ, and the MMQ Internal Strategy subscale was also correlated positively with the Anxiety subscale. There was no correlation between anxiety, age, and the MMQ External Strategy subscale.

We found a significant moderate negative association between the DASS-21 Stress subscale and MMQ Satisfaction, MMQ Ability subscales, and MoCA total score which showed a significant negative correlation with stress. Education also demonstrated a weak negative correlation with stress. CFQ, MMQ Internal Strategy, and MMQ External Strategy subscales were correlated positively with the Stress subscale. No correlation was found between stress and age. Results are presented in Table 2.

Appendix 2. Correlation between mood, demographic variables, subjective memory complaints, cognitive status

|  | DASS-21 Depression | DASS-21 Anxiety | DASS-21 Stress |
| --- | --- | --- | --- |
| Age | -0.050 | -0.111 | -0.092 |
| Education | -0.446** | -0.314** | -0.321** |
| CFQ | 0.486*** | 0.433*** | 0.407*** |
| MMQ Satisfaction | -0.495*** | -0.373*** | -0.444*** |
| MMQ Ability | -0.381*** | -0.342** | -0.357*** |
| MMQ External Strategy | 0.016 | 0.019 | 0.130** |
| MMQ Internal Strategy | 0.411*** | 0.295*** | 0.419*** |
| MoCA total score | -0.406*** | -0.228* | -0.267* |

Note. **p* < 0.05, *p*** < 0.01, *p**** < 0.001, *df* = 78, CFQ = Cognitive Failure Questionnaire, MoCA = Montreal Cognitive Assessment, DASS-21 = Depression, Anxiety, Stress Scale-21, MMQ = Multifactorial Memory Questionnaire, External Str. = External Strategy subscale, Internal Str. = Internal Strategy subscale.

Appendix 3. Correlation between subjective, objective memory and demographic variables

Significant weak negative correlation was present between CFQ and age (*r*(78) = -0.269, *p* = 0.016), although age did not correlate with any of the MMQ subscales (MMQ Satisfaction: *r*(78) = 0.166, *p* = 0.141, MMQ Ability: *r*(78) = 0.117, *p* = 0.300, MMQ Internal Strategy: *r*(78) = 0.011, *p* = 0.919, MMQ External Strategy: *r*(78) = 0.037, *p* = 0.747). Gender did not show any significant correlation with CFQ (*r*(78) = -0.016, *p* = 0.888) and MMQ subscales (MMQ Satisfaction: *r*(78) = 0.051, *p* = 0.653, MMQ Ability: *r*(78) = 0.164, *p* = 0.145, MMQ Internal Strategy: *r*(78) = -0.053, *p* = 0.640, MMQ External Strategy: *r*(78) = 0.099, *p* = 0.382). We failed to find association between education and CFQ *(r*(78) = .0.088, *p* = 0.438) and MMQ subscales (MMQ Satisfaction: *r*(78) = 0.138, *p* = 0.221, MMQ Ability: *r*(78) = 0.001, *p* = 0.993, MMQ Internal Strategy: *r*(78) = -0.184, *p* = 0.102, MMQ External Strategy: *r*(78) = 0.037, *p* = 0.741).

MoCA did not correlated with CFQ (*r*(78) = 0.047, *p* = 0.678) and none of the MMQ subscales (MMQ Satisfaction: *r*(78) = -0.027, *p* = 0.815, MMQ Ability: *r*(78) = -0.024, *p* = 0.836, MMQ Internal Strategy: *r*(78) = -0.108, *p* = 0.338, MMQ External Strategy: *r*(78) = 0.097, *p* = 0.390).
